# Patient perspectives of a diagnosis of myeloproliferative neoplasm in a case control study

**DOI:** 10.1186/s40164-016-0043-4

**Published:** 2016-05-26

**Authors:** Mary Frances McMullin, Glen James, Andrew S. Duncombe, Frank de Vocht, Lin Fritschi, Mike Clarke, Lesley A. Anderson

**Affiliations:** 1 Centre for Cancer Research and Cell Biology, Queen’s University Belfast, Belfast, Northern Ireland, UK; 2 Centre for Public Health, Queen’s University Belfast, Belfast, Northern Ireland, UK; 3 Department of Haematology, University Hospitals Southampton NHS Foundation Trust, Southampton, Hampshire UK; 4 School of Social and Community Medicine, University of Bristol, Bristol, UK; 5 School of Public Health, Curtin University, Perth, Australia; 6 Department of Haematology, Belfast City Hospital, Queen’s University Belfast, C Floor, Lisburn Road, Belfast, BT9 7AB Northern Ireland, UK

**Keywords:** Myeloproliferative neoplasms, Classification, Case–control study, Patient perception

## Abstract

**Background:**

Myeloproliferative neoplasms (MPNs) including the classic entities; polycythemia vera (PV), essential thrombocythemia (ET) and primary myelofibrosis are rare diseases with unknown aetiology. The MOSAICC study, is an exploratory case–control study in which information was collected through telephone questionnaires and medical records.

**Methods:**

As part of the study, 106 patients with MPN were asked about their perceived diagnosis and replies correlated with their haematologist’s diagnosis. For the first time, a patient perspective on their MPN diagnosis and classification was obtained. Logistic regression analyses were utilised to evaluate the role of variables in whether or not a patient reported their diagnosis during interview with co-adjustment for these variables. Chi square tests were used to investigate the association between MPN subtype and patient reported categorisation of MPN.

**Results:**

Overall, 77.4 % of patients reported a diagnosis of MPN. Of those, 39.6 % recognised MPN as a ‘blood condition’, 23.6 % recognised MPN as a ‘cancer’ and 13.2 % acknowledged MPN as an ‘other medical condition’. There was minimal overlap between the categories. Patients with PV were more likely than those with ET to report their disease as a ‘blood condition’. ET patients were significantly more likely than PV patients not to report their condition at all. Patients from a single centre were more likely to report their diagnosis as MPN while age, educational status, and WHO re-classification had no effect.

**Conclusions:**

The discrepancy between concepts of MPN in patients could result from differing patient interest in their condition, varying information conveyed by treating hematologists, concealment due to denial or financial concerns. Explanations for the differences in patient perception of the nature of their disease, requires further, larger scale investigation.

## Background

Myeloproliferative neoplasms (MPNs) including polycythemia vera (PV), essential thrombocythemia (ET) and primary myelofibrosis (PMF) are rare, heterogeneous diseases with a reported pooled incidence rate of 0.84, 1.03 and 0.47 for PV, ET and PMF, respectively [1]. MPNs are characterised by an overproduction of mature and immature cells in one or more cell types within the myeloid lineage. Overall survival of PV and ET is respectable with median survival of 10–16 years for PV [2] and 10–22 years for ET [2, 3]. PMF confers much poorer median survival of 4–5 years [4]. These figures reflect the high prevalence of PV and ET compared to PMF in the general population [1]. In 2008, the World Health Organisation (WHO) officially changed the classification of these conditions to neoplastic due to their demonstrated acquired clonality [5]. MPNs possess great genetic complexity which contributes to disease pathogenesis and outcome [6]. Major advances in understanding the genetic complexity of these diseases first came in 2005 when the Janus Kinase 2 (*JAK2*) V617F acquired mutation was discovered, altering diagnostic classification and leading to new treatments for MPNs [7]. Further acquired abnormalities in MPN were the discovery of mutations in the thrombopoietin receptor (*MPL*) [8] in 2006 and calreticulin (*CALR*) in 2013, further revolutionising our understanding, diagnostic classification, treatment and prognosis of MPNs [9, 10].

The aetiology of these diseases remains elusive with limited information available on potential risk factors. A systematic review conducted by Anderson et al. identified some medical, environmental and lifestyle risk factors to be associated with MPNs [11]. Registration and collection of MPN data has been limited. Cancer registries, for example in the UK and USA with adequate healthcare resources, did not begin a comprehensive collection of MPN data until very recently due to the change in classification of these diseases to cancer [12–14]. Additionally, in countries with limited healthcare resources, diagnostic accuracy and registration of MPNs, particularly in the *JAK2* era, where genetic testing is not feasible, may contribute to misclassification and under-reporting of MPNs [15–17].

The unknown aetiology of MPNs justified the need for conducting a pilot exploratory case–control study, to firstly ascertain optimal methodology for a larger UK-wide study, and secondly to collect information to identify potential risk factors associated with MPNs. The long survival and high prevalence of MPNs, particularly PV and ET, has permitted individuals to experience changes in clinical classification of their disease over time. Understanding of true MPN classification may be unknown to patients because of this and other factors. As part of the study on epidemiology and quality of life we were able to collect clinician derived data regarding patients MPN diagnosis, and we aimed to assess patient perception of their disease nomenclature and classification.

## Results

Overall, 106 MPN patients (37 PV, 55 ET and 14 PMF) participated in the study. Patient characteristics are presented in Table 1. Patient reported treatments were aspirin (76), hydroxycarbamide (13) trial (3) interferon alpha (3) anagrelide (1), venesection (3), danazol (1) and erythropoietin (1).

**Table 1:** Characteristics of MPN patients

	Polycythemia vera	Essential thrombocythemia	Primary myelofibrosis
Location			
Southampton	24 (64.9 %)	31 (56.4 %)	11 (78.6 %)
Belfast	13 (35.1 %)	24 (43.6 %)	3 (21.4 %)
Gender			
Male	13 (35.1 %)	19 (34.6 %)	10 (71.4 %)
Female	24 (64.9 %)	36 (65.5 %)	4 (28.6 %)
Age (years)			
Mean (SD)	62.1 (13.2)	63.2 (13.4)	66.9 (4.2)
Education			
Pre-University	25 (67.6 %)	37 (67.3 %)	12 (85.7 %)
University	12 (32.4 %)	16 (29.1 %)	2 (14.3 %)
Did not report	0	2 (3.6 %)	0

Remarkably, only 76.4 % of patients reported their diagnosis as MPN to any of the 3 questions during the telephone interview with 39.6 % of patients recognising MPN as a ‘blood condition’, 23.6 % as ‘cancer’, and 13.2 % acknowledged their MPN as an ‘other condition’ see Table 2. Only one patient recorded both ‘blood condition’ and ‘cancer’ and there was no other overlap between categories.

**Table 2:** Patient recall of mpn diagnosis and classification

MPN subtype^a^	Patient reporting of MPN in the telephone questionnaire				Total
	Did not report	Blood condition	Cancer	Other condition	
PV	5 (13.5 %)	21 (56.8 %)	6 (16.2 %)	5 (13.5 %)	37
ET	18 (32.7 %)	16 (29.1 %)	15 (27.3 %)	6 (10.9 %)	55
PMF	2 (14.3 %)	5 (35.7 %)	4 (28.6 %)	3 (21.4 %)	14
Total	25 (23.6 %)	42 (39.6 %)	25 (23.6 %)	14 (13.2 %)	106

^a^As recorded on the medical proforma completed by the patients consultant hematologist

ET patients were significantly more likely than PV patients not to report their condition at all (*p* = 0.037) and significantly more likely than PV and PMF patients combined (*p* = 0.021).

Patients with PV were more likely than those with ET, but not PMF, to report their disease as a ‘blood condition’ (*p* = 0.039 and *p* = 0.943, respectively). A similar proportion of ET and PMF patients reported their condition as a ‘blood condition’ (*p* = 0.835).

There were no significant differences in the proportion of patients reporting their condition as ‘cancer’ (PV vs. ET, *p* = 0.215; PV vs. PMF, *p* = 0.321 and ET vs. PMF, *p* = 0.923). Similarly, there were no differences in reporting as ‘other condition’ (PV vs. ET, *p* = 0.706; PV vs. PMF, *p* = 0.488 and ET vs. PMF, *p* = 0.297).

When adjusted for potential confounding variables of MPN subtype, age, gender and educational status, cases from Southampton were highly significantly more likely than cases from Belfast to report their diagnosis as MPN at interview (OR 5.51 95 % CI 1.95–15.53, *p* = 0.001). Age and education level did not affect whether or not patients reported their diagnosis during the interview (*p* = 0.365 and *p* = 0.448, respectively).

Reporting of MPN as a ‘cancer’ by patients diagnosed before or after the WHO classification change in 2008 did not significantly differ (18.4 vs. 28.1 %, *p* = 0.241). There were also no significant differences in reporting MPN as a ‘blood condition’ (38.8 vs. 40.4 %, *p* = 0.869), an ‘other condition’ (16.3 vs. 10.5 %, *p* = 0.379), or not at all (26.5 vs. 21.1 %, *p* = 0.508) before or after 2008.

## Discussion

To our knowledge, this is the first time that assessment of MPN classification from a patient perspective has been ascertained. In a group of patients who had volunteered for the study on the basis of their diagnosis, the variable reporting of what they believe their condition to be is intriguing. However, no major factors could be identified which may shed any light on these variations. There were no significant differences in reporting diagnosis by age, gender, or level of education.

We did observe some differences in perceptions between MPN subtypes. Patients with PV were more likely to report their disease as a ‘blood condition’ than those with ET. This may be due to many patients with PV requiring regular venesections acting as a prompt to the nature of the disorder. Likewise, the finding that patients with ET were the least likely to report any relevant condition may be due to their receiving less treatment intervention overall than other MPN subtypes.

A particular issue for MPN patients and clinicians has been the WHO definition change from Myeloproliferative disorders (MPD) to myeloproliferative neoplasms (MPN) in 2008 due to the acquired clonal nature of the disorders leading to their new definition as a form of cancer. This change took time to filter through to clinicians and patients who may not be willing to reclassify their initial diagnosis. The reclassification provoked a lot of controversy in patient forums and this may have influence in the wider patient arena. Interestingly, our data show that there was no significant difference between the cohorts diagnosed before or after 2008 in the proportion of patients volunteering their diagnosis as cancer despite this change occurring over 7 years ago. This may reflect a reluctance to label MPN as cancer on the part of hematologists as well as patients and this merits further study.

We observed that patients recruited from Southampton were 5 times more likely to report their diagnosis as MPN than patients recruited from Belfast. That this effect was independent of educational status is highly suggestive that the difference may be in the way the diagnosis has been imparted. Clinicians may explain diagnoses to patients in different formats for a variety of reasons including truth telling custom and varying cultural practices [18]. There may be differences in patient and clinician reports on diagnostic information conveyed [19, 20] with clinicians perhaps avoiding using the word cancer due to the distress this can cause.

We did not identify any differences in reporting of disease by gender, age or educational level which is perhaps a little surprising. Certainly in some other studies older age and higher levels of education are associated with more awareness of a terminal cancer diagnosis [21]. Older patients have also been seen to be more likely to deny a cancer diagnosis [22]. Recently diagnosed patients will have had access to a greater range of patient information from both health care professionals, patient support groups and the internet yet we have not found here any greater awareness in those recently diagnosed.

Moreover patients have different personalities and different coping structures. This may impact on how they perceive their diagnosis and information given to them [23] and of course denial is one particular coping structure for some cancer patients [22]. Furthermore, some patients may have contrasting priorities in health seeking behaviour with some gathering more information outside of the clinical setting than others. Finally, not acknowledging MPN diagnosis or not classifying MPN as a cancer could reflect patient concerns about perceived financial and insurance disadvantages of full disclosure.

It needs to be recognised that this group of MPN patients were being asked to volunteer their diagnosis over the telephone. Different replies may have been obtained if patients were asked to recognise their diagnosis from a menu choice provided by the interviewer. Furthermore, those patients who volunteered a diagnosis of MPN were not subsequently questioned specifically as to whether they recognised this as a form of cancer in view of the sensitivity surrounding this issue.

Further larger scale investigation to address these remaining ambiguities in MPN classification from a patient viewpoint is warranted.

## Methods

The pilot MOSAICC (myeloproliferative neoplasms: an in-depth case–control) study was a feasibility study which recruited 106 MPN patients and 127 controls. MPN patients were recruited from two sites: Belfast City Hospital, Belfast, Northern Ireland and University Hospital Southampton NHS Foundation Trust, Southampton, England. Eligible patients were identified by their consultant hematologist (MFMcM, Belfast and ASD, Southampton) and classified according to the WHO diagnostic criteria [5]. Interviews were conducted by a biomedical scientist. Information was ascertained from participants through a structured telephone administered questionnaire and information sheets which included demographic, medical history, residence history, occupation, life style and quality of life (QoL) questions. Interviews lasted 45 min on average. Information on gender and age at the time of interview were obtained. Level of education was obtained and patients classified as having pre- or post-University education.

In relation to medical history patients were asked “Have you been diagnosed with any blood conditions?”, “Have you ever been diagnosed with cancer?” and “Before a year ago did a doctor ever tell you that you had any other medical condition?” in sequence. If the patient responded ‘yes’ to any of these questions further details were obtained about the condition, date of diagnosis and treatment and were transcribed using the patient’s own language. Exact details of patient MPN sub-type, year of diagnosis and treatment regimens were collected through use of a proforma via access to medical records by their treating hematologist. Ethical approval was obtained from the Office for Research Ethics Committee, Northern Ireland (OREC-NI) and the study registered on the NIHR portfolio in England and Wales.

### Statistical analysis

Crude and adjusted logistic regression analyses were utilised to evaluate the role of gender, age (mean and SD), education (pre-/post-University) and location (Southampton vs. Belfast), in whether or not a patient reported their diagnosis during interview with co-adjustment for these variables. Chi square tests were used to investigate the association between MPN subtype and patient reported categorisation of MPN (‘blood condition’, ‘cancer’, ‘other condition’).

## Abbreviations

CALR: calreticulin
ET: essential thrombocythemia
JAK2: Janus kinase 2
MOSAICC: myeloproliferative neoplasms: an in-depth case–control study
MPL: thrombopoietin receptor
MPNs: myeloproliferative neoplasms
PV: polycythemia vera
QoL: quality of life
WHO: World Health Organisation

## References

[1] Titmarsh GJ, Duncombe AS, McMullin MF (2014). How common are myeloproliferative neoplasms? a systematic review and meta-analysis. Am J Hematol.

[2] Hoffbrand AV, Moss PAH, Pettit JE (2006). Essential haematology myeloproliferative disorders.

[3] Passamonti F, Rumi E, Pungolino E (2004). Life expectancy and prognostic factors for survival in patients with polycythemia vera and essential thrombocythemia. Am J Med.

[4] Cervantes F, Dupriez B, Passamonti F (2012). Improving survival trends in primary myelofibrosis: an international study. J Am Soc Clin Oncol.

[5] Vardiman JW, Porwit A, Brunning RD, et al. Introduction and overview of the classification of myeloid neoplasms. In: Swerdlow SH, Campo E, Harris NL, et al., editors. The World Health Organization (WHO) classification of tumours of haematolpoeitic and lymphoid tissues. 4th ed. Lyon: IARC; 2008. p. 18–30.

[6] Tefferi A (2010). Novel mutations and their functional and clinical relevance in myeloproliferative neoplasms: JAK2, MPL, TET2, ASXL1, CBL, IDH and IKZF1. Leukemia.

[7] Levine RL, Wadleigh M, Cools J (2005). Activating mutation in the tyrosine kinase JAK2 in polycythemia vera, essential thrombocythemia, and myeloid metaplasia with myelofibrosis. Cancer Cell.

[8] Pikman Y, Lee BH, Mercher T (2006). MPLW515L is a novel somatic activating mutation in myelofibrosis with myeloid metaplasia. PLoS Med.

[9] Nangalia J, Massie CE, Baxter EJ (2013). Somatic CALR mutations in myeloproliferative neoplasms with nonmutated JAK2. N Engl J Med.

[10] Klampfl T, Gisslinger H, Harutyunyan AS (2013). Somatic mutations of calreticulin in myeloproliferative neoplasms.

[11] Anderson AL, Duncombe AS, Hughes M (2011). Environmental, lifestyle, and familial/ethnic factors associated with myeloproliferative neoplasms. Am J Hematol.

[12] Bagguley T, Roman E, Bolton E, Oliver S. Haematological malignancies and cancer registration in England quality appraisal comparing data from the national cancer data repository (NCDR) with the population-based Haematological Malignancy Research Network (HMRN). 2012.

[13] Selinger HA, Ma X (2009). Jakking up tumor registry reporting of the myeloproliferative neoplasms. Am J Hematol.

[14] Rollison DE, Howlader N, Smith MT (2008). Epidemiology of myelodysplastic syndromes and chronic myeloproliferative disorders in the United States, 2001–2004, using data from the NAACCR and SEER programs. Blood.

[15] López-Gómez M, Malmierca E, de Górgolas M, Casado E (2013). Cancer in developing countries: the next most preventable pandemic. The global problem of cancer. Crit Rev Oncol Hematol.

[16] Arkin DMP, Ray FB, Erlay JF, Isani PP (2001). Mini review estimating the world cancer burden. Globocan.

[17] Jemal A, Bray F, Center MM (1999). Global cancer statistics. CA Cancer J Clin.

[18] Mystakidou K, Parpa E, Tsilika E, Katsouda E, Vlahos L (2004). Cancer information disclosure in different cultural contexts. Support Care Cancer.

[19] Mosconi P, Meyerowitz BE, Liberati MC (1991). Disclosure of breast cancer diagnosis: patient and physician reports. Ann Oncol.

[20] Jenkins V, Solis-Trapala I, Langridge C, Catt S, Talbot DC, Fallowfield LJ (2011). What oncologists believe they said and what patients believe they heard: an analysis of phase 1 trial discussions. J Clin Oncol.

[21] Corli O, Apolone G, Pizzuto M, Cesaris L, Cozzolino, Orsi L, Enterri L (2009). Illness awareness in terminal cancer patients: an Italian study. Palliat Med.

[22] Vos MS, de Haes JCJM (2007). Denial in cancer patients, an explorative review. Psycho-Oncology.

[23] Husson O, Denollet J, Oerlemans S, Mols F (2013). Satisfaction with information provision in cancer patients and the moderating effect of type D personality. Pscho-Oncology.

